# Glycerinated Calf Lymph

**Published:** 1897-06-12

**Authors:** 


### GLYCERINATED CALF LYMPH.

In answer to a question recently asked by Sir W. Priestley in the House of Commons, Mr. T. W. Russell held out some hope that it might be practicable to issue gratuitous supplies of glycerinated calf lymph to all registered medical practitioners applying for it. This opens up many wide and important considerations. If this official answer is to be taken as indicating the probable drift of the legislation which must of necessity be shortly introduced, we can at once see what a complete change in the whole mechanism of the Vaccination Acts is likely to take place. The existing arrangements are made on the understanding that arm to arm vaccination is always to be aimed at, and that the gratuitous supply of lymph granted by the National Vaccine Establishment is to be made use of only for the starting of a series of arm to arm cases. Hence to a large extent the appointment of public vaccinators. To some degree their utility arises from the greater skill which must always come from practice in the performance even of so small an operation as vaccination, but the main and preponderating reason for their existence is to be found in the necessity of providing each vaccinator with a sufficiently large supply of cases to enable him to maintain a series. Once admit, however, that vaccination can be done efficiently by glycerinated calf lymph, and once give a free supply of this substance to he profession, and the necessity for the public vaccinator will be greatly lessened, and that for the public vaccinating stations, with all its evils, will absolutely vanish. It has to be admitted that to a considerable extent the revolt against vaccination has been a revolt against officialism, and that it has been greatly stimulated by the natural dislike felt by every parent to that intimate relationship with other children which to the mother's mind seems to be implied in the immediate transmission of lymph from child to child. Let vaccination be done by the family doctor; let it be performed by aid of a product from the laboratory (although a pathological one) instead of by means of a crude and obviously diseased secretion from another child, and let there be no longer any mixing of babies, and we believe vaccination will again obtain that support and recognition among the masses of the people that it always has received among the more educated classes of the community.

